# The long-term impact of immediate verbal feedback of hand hygiene compliance after overt observation sessions, as assessed by continuous closed-circuit television monitoring in an intensive care setting

**DOI:** 10.1186/s13690-022-00887-2

**Published:** 2022-05-18

**Authors:** Ilana Livshiz-Riven, Hovav Azulay, Leonid Koyfman, Alex Gushanski, Seada Askira, Vered Ischa Abar, Benjamin F. Gruenbaum, Evgenia Ivanov, Moti Klein, Asaf Danziger, Ronit Nativ, Abraham Borer, Tomer Ziv-Baran, Evgeni Brotfain

**Affiliations:** 1 Quality Unit, Soroka Medical Center, Faculty of Health Science, Ben-Gurion University of the Negev, Beer Sheva, Israel; 2 Department of Nursing, Faculty of Health Science, Ben Gurion University of the Negev, Beer Sheva, Israel; 3 Department of Internal Medicine C, Soroka Medical Center, Faculty of Health Science, Ben-Gurion University of the Negev, Beer Sheva, Israel; 4 Department of Anesthesiology and Critical Care, General Intensive Care Unit, Soroka Medical Center, Faculty of Health Science, Ben-Gurion University of the Negev, Beer Sheva, Israel; 5 Infection Control Unit, Soroka Medical Center, Faculty of Health Science, Ben-Gurion University of the Negev, Beer Sheva, Israel; 6 Department of Anesthesiology and Perioperative Medicine, Mayo Clinic, Jacksonville, FL USA; 7 Department of Internal Medicine A, Soroka Medical Center, Faculty of Health Science, Ben-Gurion University of the Negev, Beer Sheva, Israel; 8 School of Public Health, Sackler Faculty of Medicine, Tel Aviv University, Tel-Aviv, Israel

**Keywords:** Closed-circuit television, Hand hygiene, Health care associated infections, Health care workers, Intensive care unit

## Abstract

**Background:**

Hand hygiene compliance by health care workers (HCWs) is pivotal in controlling and preventing health care associated infections. The aim of this interventional study is to assess the long-term impact of personal verbal feedback on hand hygiene compliance of HCWs in an intensive care unit (ICU) immediately after overt observation by an infection control nurse.

**Methods:**

An infection control nurse overtly observed HCWs’ hand hygiene compliance and immediately gave personal verbal feedback with emphasis on aseptic technique. Overt non-interventional sessions were also performed. We measured compliance rates using covert continuous closed-circuit television (CCTV) monitoring. We compared these rates to previously-published hand hygiene compliance data.

**Results:**

Overall compliance rates in the first (41.5%) and third phases (42%) of the study, before and after the intervention were similar. The two moments that were lowest in the first phase, “before aseptic contact” and “after exposure to body fluids”, showed significant improvement, but two moments showed a significant decline in compliance: “before patient contact” and “after contact with patient surrounding”. The compliance rates during the intervention phase were 64.8% and 63.8% during the sessions with and without immediate verbal personal feedback, respectively.

**Conclusion:**

The overall hand hygiene compliance rate of HCWs did not show an improvement after immediate verbal personal feedback. Covert CCTV observational sessions yielded much lower hand hygiene compliance rates then overt interventional and non-interventional observations. We suggest that a single intervention of personal feedback immediately after an observational session is an ineffective strategy to change habitual practices.

## Background

Health care associated infections are a major concern in maintaining patient safety, and the prevention and containment of infections should be prioritized in hospital settings. In developed countries, health care associated infections can affect 5–15% of hospitalized patients, including up to 37% of those admitted to the intensive care unit (ICU) [1, 2]. The potential long-term effects of health care associated infections include prolonged hospital stays, long-term disability, increased resistance of microorganisms to antimicrobials, financial burden, higher incidence of mortality, and emotional stress on patients and their families [3]. The risk of acquiring health care associated infections depends on factors related to the infectious agent, including virulence, capacity to survive in the environment, and antimicrobial resistance; the host, including advanced age, low birth weight, underlying diseases, state of debilitation, immunosuppression, and malnutrition; and the environment, including ICU admission, prolonged hospitalization, invasive devices and procedures, and antimicrobial therapy [3]. Transmission of health care associated pathogens takes place through direct and indirect contact, droplets, air and a common vehicle [4]. Transmission through the hands of contaminated health care workers (HCWs) hands is the most common transmission pattern in most hospital settings.

Immediate feedback and interventional measures for HCWs during hand hygiene monitoring is a crucial part of maintaining hygiene [5]. Several types of interventions to improve hand hygiene have been described in the literature. Most studies demonstrated that multifaceted approaches promoted hand hygiene compliance more effectively than single interventions [6, 7]. It was subsequently theorized that the more useful interventions involved a combination of written material with reminders and continuous feedback on performance, as opposed to interventions involving novel equipment (such as automated sinks or moisturized soaps) [7, 8]. Auditing and immediate, real-time feedback are widely-used strategies to improve professional practice [9–12]. The format of real-time feedback can include verbal, written, or a combination of written and verbal responses by a team of observers [10].

In the ICU at Soroka Medical Center, in Beer Sheva, Israel, a new method of hand hygiene control involving the use of continuous daily closed-circuit television monitoring (CCTV) was developed in 2017. Between 2017–2020, there have been two methods of hand hygiene monitoring: an overt technique performed by trained observers who manually observe and report hand hygiene activities, and a covert technique using a CCTV monitoring system. In a previously published study, CCTV was determined to be a reliable and objective method of hand hygiene control for HCWs [13]. Moreover, the CCTV method has several advantages, especially the neutralization of the Hawthorne effect, in which participants alter their behavior when they know they are being observed. In the present study, we analyzed the effect of immediate verbal feedback on HCWs hand hygiene habitual practices, using overt observation, and CCTV monitoring.

## Aim

The aim of this study was to analyze the efficacy of real-time, personal verbal feedback to HCWs about their hand hygiene using two methods, overt observations and covert monitoring with CCTV.

## Methods

### Study design

This was an interventional, prospective and correlational study performed at a general ICU. Measurements were conducted in three chronological phases. The first phase of the study, before the intervention, included hand hygiene compliance rate observations using covert CCTV monitoring throughout 2017. The second phase (January 2018–May 2018) included immediate personal verbal feedback after an overt observation session performed by an infection control nurse. In addition, overt observations without interventions were carried out by infection control personnel. The third phase (July 2019–June 2020) involved documentation of CCTV monitoring of HCWs without intervention.

### Setting

Soroka Medical Center is a 1150-bed tertiary-care university teaching hospital located in southern Israel and serving a population of over 500,000 individuals. Our general ICU has sixteen beds and is staffed by six physicians and forty nurses and auxiliary medical staff.

### Inclusion criteria

The study population incorporated all the general ICU HCWs, including staff physicians, registered nurses and auxiliary workers.

### Exclusion criteria

HCWs who were not involved in the treatment of critically-ill general ICU patients were excluded from the study.

### Study protocol

A team of five trained observers (comprised of three physicians, one registered ICU nurse and one infection control nurse) oversaw the study design and implementation. We have previously described the methods employed for training the observers and validating their findings [13]. For phase 1, the current study utilizes previously published data on CCTV observations conducted and validated in 2017 [13]. In this phase, the observers did not perform any immediate feedback at the end of the observations. As a mandatory regulation by the Ministry of Health in Israel, the infection control unit routinely conducted overt observations and collected data regarding HH compliance among HCWs. Hand hygiene data pertaining to the HCWs in the ICU was sent to the unit management every several months.

This was followed by the second intervention phase. In addition to the reports submitted to ICU management regarding the overall compliance rates every several months, infection control nurses (SA and VA) conducted personal feedback sessions immediately after each overt observation session to the HCWs whom they observed. This feedback was specific to each HCW, and included an opportunity to educate the HCW on how to perform hand hygiene properly, particularly emphasizing the importance of aseptic technique. Additional simultaneous non-synchronized overt observations were conducted during the same months of intervention. For this group, other staff in the infection control unit observed HCWs overtly but without providing intervention.

The third phase included covert CCTV observation (performed by HA and AD). As in the first phase, the team observed one or two HCWs at a time. In each phase, more than 200 opportunities to perform hand hygiene were observed in 20 ± 10-min-long sessions. These sessions were observed for five moments for hand hygiene as recommended by the World Health Organization (WHO). These five moments include: 1) before patient contact, 2) before aseptic contact, 3) after exposure to body fluids, 4) after patient contact, and 5) after contact with patient surrounding [1].

### Observational tools

The observational data recorded by both the overt observations and by CCTV were registered on an observation documentation report form based on the WHO recommendations that was issued by the Infection Control Unit at Soroka Medical Center. When more than one moment of hand hygiene was observed, it was assigned to the first indication that was detected.

### Statistical analysis

The chi-squared test was performed to compare proportions of HH compliance in non-synchronized overt observations. The Wald Z-Test with Continuity Correction test was performed to compare covert observations phases. Statistical analysis was performed using NCSS software (NCSS 2020 Statistical Software, 2020, NCSS, LLC. Kaysville, Utah, USA, ncss.com/software/ncss.). A *p*-value of < 0.05 was considered significant.

### Ethical approval

The Human Research and Ethics Committee at the Soroka Medical Center approved this study (0373–16-SOR). All participants were aware of the possibility of being observed for HH compliance via CCTV. The footage from CCTV was not recorded.

## Results

The results of hand hygiene compliance rates among HCW in the ICU using CCTV from 2017 have been previously published [13]. This data was used as the baseline for HH compliance rates for the control portion of our study (Table 1). Phase 2 of the study, the intervention, was conducted from January 2018 to May 2019 and included 213 opportunities to perform hand hygiene with corresponding educational feedback. Simultaneously, 495 additional opportunities were observed by non-synchronized overt observers from the infection control unit, without intervention (Table 2). There was no difference found between overt observations with immediate intervention and the overt non-synchronized observations during Phase 2 of the study, with moderate hand hygiene compliance among the HCWs at 64.8% and 63.8% respectively (Table 2).

**Table 1:** Comparison of hand hygiene compliance as measured by covert observations using CCTV in Phase 1 before intervention and in Phase 3 after intervention, using the 5-moment model

	Covert observations		Relative Risk (Lower & Upper CI)	*P*-Value
	Phase 1	Phase 3		
	2017	2019–2020		
Before patient contact	18/33 (54.5%)	80/245 (32.7%)	1.67 (1.17, 2.39)	0.0228^**^
Before aseptic contact	7/54 (13%)	16/31 (51.6%)	0.25 (0.12, 0.54)	0.0003^*^
After exposure to body fluids	30/122 (24.6%)	33/69 (47.8%)	0.51 (0.35, 0.76)	0.0018^*^
After patient contact	48/70 (68.6%)	119/202 (58.91%)	1.16 (0.96, 1.42)	0.1976
After contact with patient surrounding	45/78 (57.7%)	46/153 (30.1%)	1.92 (1.41, 2.61)	0.0001^**^
Total of hand hygiene compliance	148/357 (41.5%)	294/700 (42%)	0.99 (0.85, 1.15)	0.9176

^*^Improvement; ^**^ Decline

**Table 2:** HCW compliance with hand hygiene guidelines during overt observations with immediate educational intervention and feedback according to the 5-moment model, and of non-interventional non-synchronized compliance rates in Phase 2 between January 2018-May 2019 (*n* = 707 opportunities for HH)

	Compliance in overt observations with immediate intervention and feedback	Compliance in overt non-interventional non-synchronized observation	*P*-Value*
	(*n* = 213)	(*n* = 494)	
Before patient contact	19/39 (48.7%)	50/96 (52.1%)	0.85
Before aseptic contact	13/31 (41.9%)	29/81 (35.8%)	0.663
After exposure to body fluids	29/41(70.7%)	81/104 (77.9%)	0.393
After patient contact	50/60 (83.3%)	98/126 (77.7%)	0.44
After contact with patient surrounding	27/42 (64.3%)	57/87 (65.5%)	1
Total opportunities	138/213 (64.8%)	315/494 (63.8%)	0.864

^*^Chi-Square test, 2-saided significance

The overall compliance of HCWs who were observed with CCTV did not improve after verbal personal feedback. During 2019, the rate of compliance was 148/357 (41.5%) and Phase 3 had a nearly-identical compliance rate (294/700, 42%, Table 1). However, there was a significant rise in hand hygiene compliance of HCWs after aseptic procedures and after bodily fluid exposures, as well as a significant fall in compliance after contact with patient surrounding (decline from 57.7% to 30.1% after interventional sessions), and before patient contact (decline from 54.5% to 32.7% after interventional sessions).

It is noteworthy that the overall compliance rates observed in overt observations are different from those observed with covert monitoring. During the second phase of the study, overall compliance in overt educational sessions was observed at 64.8% compared to approximately 42% overall compliance in covert post-intervention observation (Table 1 and 2). This change may be explained by the Hawthorne effect on HCWs who are aware of being observed [13].

Additional overt sessions on hand hygiene compliance without intervention were performed during Phase 2 between January 2018 and May 2019. There was no difference in HH compliance rates in non-interventional non-synchronized observation compared to overt observations with immediate verbal intervention (63.8% to 64.8% respectively, Table 2).

## Discussion

Most health care associated infections are spread by direct contact, especially by HCWs’ hands [3–7]. Accordingly, HH has traditionally been considered the most important means of preventing health care associated infections. Transmission of micro-organisms from the hands of HCWs to a patient or to the environment can be prevented either by mechanical removal such as washing with soap and water or an aqueous antiseptic (e.g. chlorhexidine gluconate) and drying, or by the use of alcohol-based hand rubs [9–12]. In 2009, the WHO published guidelines for implementing and evaluating hand hygiene programs in healthcare settings [1]. They identified five interventional methods to be implemented: alcohol-based hand rubs at point of care or carried by the healthcare worker, training and education, observation and performance feedback, reminders (such as posters), and administrative support/institutional safety climate [11, 12].

Education is an important component of hand hygiene interventions [11, 12]. Information, usually based on the WHO guidelines issued in 2009 [1], is displayed on posters and flyers. E-learning materials and simulation, as well as lectures and workshops, have been implemented as educational resources [13]. Teaching is usually delivered by in-house infection prevention teams or external consultants who perform outreach to clinical areas. Some studies of compliance refer to hand hygiene reminders in various forms [13–15]. Commonly, audits are performed with performance feedback given to wards, units, organizations and, occasionally, to individuals. In some studies, individual verbal as well as written feedback is given, and there may be a graphic display of hand hygiene audit findings in clinical areas with high infection rates [15]. Despite multifaceted interventions to improve hand hygiene compliance, the most effective method remains unclear.

In previously published literature, different interventional strategies for improvement of hand hygiene compliance of HCWs has been described. Wiedenmayer et al. evaluated the impact of hand hygiene training as part of a water, sanitation and hygiene program [14]. They demonstrated that the overall level of hand hygiene after a combined water, sanitation and hygiene program and training intervention showed improvement from inadequate to basic. Villarreal et al. found that oral educational intervention substantially increased visitors' hand hygiene compliance rate [16]. A systematic review published by Seo et al. showed that multimodal or dual interventions including education, monitoring and providing feedback, campaigns, and cues effectively improved hand hygiene compliance of HCWs [15]. Anwar et al. provided an interventional educational program for improvement of hand hygiene compliance of HCWs in six ICUs [17]. They found a significant increase of overall hand hygiene compliance from 30.9 (95% CI: 27.2–34.6%) before intervention to 69.5 (95% CI: 65.2–72.6%) post intervention. Importantly, their interventional educational program included a multimodal approach of usage workplace posters and explanatory leaflets depicting the 5 moments for hand hygiene, as well as instructions and reminders to use hand sanitizers and wash hands. In addition, active presentations, videos and training handouts were given to each participant [15].

In the present study, we conducted personal verbal feedback sessions, as well as education and an opportunity to perform hand hygiene, to the HCWs immediately after each overt observation session. Overall hand hygiene compliance rate had no significant change in covert sessions after verbal interventional sessions (Table 1). This may be attributable to the relatively small numbers of interventional sessions, with only 213 opportunities to perform hand hygiene after verbal intervention.

Additionally, the last phase of our study was performed during the COVID-19 pandemic. In previously published literature [18–22], HCWs demonstrated both increased and decreased hand hygiene compliance rates during different phases of the pandemic. Huang et al [18] reported that HCW hand hygiene rate on room entry decreased over time; on room exit, it increased by 13.73% during the first wave of COVID-19, decreased by 9.87% during the post-lockdown period, then rebounded by 2.82% during the second wave of the epidemic. Makhni et al [19] also demonstrated statistically significant increases and decreases of hand hygiene compliance rates monthly during the pandemic period in 2020. In response, Sandbekken et al [21] suggested that there are many factors that influence hand hygiene adherence, including education, occupation status and glove use. These discrepancies in hand washing may be attributable to the many uncertainties about COVID-19, especially when it first emerged. In our study, we suggest that the absence of the overall effect of our interventions might coincide with this period of fluctuation due to the pandemic.

However, we demonstrated sustained increase in compliance rate in two moments of hand hygiene (before aseptic contact and after exposure to body fluids), and a decrease in two moments (after contact with patient surrounding and before patient contact). The individual feedback emphasized the need for hand hygiene before aseptic procedures. It is likely that this feedback played a role in how HCWs performed hand hygiene afterwards. It seems to explain that the focus shifted from “before patient contact” and the delivery of the individual feedback likely had an effect.

Also, no difference in compliance rates were observed in overt observations with immediate intervention and feedback compared to non-interventional, non-synchronized observations (Table 2). In contrast to previous studies that incorporated multiple interventional approaches, in this present study, we used only one verbal intervention. This difference might explain the insignificant impact of interventional measures on hand hygiene compliance rate of HCWs in both overt and covert observational sessions. These findings correlate well with a previously published study by Pires et al. [18], which also identified resistance to change in hand hygiene compliance among HCWs.

Our study had a few limitations. We had a low rate of occurrence of hand hygiene compliance assessment after verbal intervention. Moreover, a one-time verbal intervention for education was used, rather than a long-term educational program. The study design also precluded the ability to evaluate sampling error because the GICU HCW personnel varied greatly during the observation period. The personnel and protocol changes that occurred during the COVID-19 pandemic may have affected the results. However, it is still surprising that a global focus on preventing infectious transmission in 2020 did not appear to skew our results toward more hand hygiene compliance.

## Conclusion

We believe that our study is crucial to developing more effective ways to combat infection in hospital settings. Since our data suggests that verbal intervention alone does not yield significant results, hand hygiene compliance using multimodal interventional models will hopefully lead to more effective and safe practices. Further research will be especially valuable as hospitals begin to reassess their daily techniques for preventing and limiting infections in the wake of the COVID-19 pandemic, which may lead to widespread institutional changes.

## Data Availability

The data that support the findings of this study are available from the corresponding author, [EB], upon reasonable request.

## Abbreviations

HCWs: Health care workers
ICU: Intensive care unit
CCTV: Continuous closed-circuit television
WHO: World Health Organization
